# Modifications to ART service delivery models by health facilities in Uganda in promotion of intervention sustainability: a mixed methods study

**DOI:** 10.1186/s13012-017-0578-8

**Published:** 2017-04-04

**Authors:** Henry Zakumumpa, Sara Bennett, Freddie Ssengooba

**Affiliations:** 1 School of Public Health, Makerere University, Kampala, Uganda; 2 Bloomberg School of Public Health, Johns Hopkins University, Baltimore, USA

**Keywords:** Sustainability, Adaptation, Implementation, ART scale-up, Health systems, Health services delivery, Resource-limited settings

## Abstract

**Background:**

In November 2015, WHO released new treatment guidelines recommending that all diagnosed as HIV positive be enrolled on antiretroviral therapy (ART). Sustaining and expanding ART scale-up programs in resource-limited settings will require adaptations and modifications to traditional ART delivery models to meet the rapid increase in demand. We identify modifications to ART service delivery models by health facilities in Uganda to sustain ART interventions over a 10-year period (2004–2014).

**Methods:**

A mixed methods approach involving two study phases was adopted. In the first phase, a survey of a nationally representative sample of health facilities (*n* = 195) in Uganda which were accredited to provide ART between 2004 and 2009 was conducted. The second phase involved semi-structured interviews (*n* = 18) with ART clinic managers of 6 of the 195 health facilities purposively selected from the first study phase. We adopted a thematic framework consisting of four categories of modifications (format, setting, personnel, and population).

**Results:**

The majority of health facilities 185 (95%) reported making modifications to ART interventions between 2004 and 2014. Of the 195 health facilities, 157 (81%) rated the modifications made to ART as “major.” Modifications to ART were reported under all the four themes. The quantitative and qualitative findings are integrated and presented under four themes. *Format*: Reducing the frequency of clinic appointments and pharmacy-only refill programs was identified as important strategies for decongesting ART clinics. *Setting*: Home-based care programs were introduced to reduce provider ART delivery costs. *Personnel*: Task shifting to non-physician cadre was reported in 181 (93%) of the health facilities. *Population*: Visits to the ART clinic were rationalized in favor of the sub-population deemed to have more clinical need. Two health facilities focused on patients living nearer the health facilities to align with targets set by external donors.

**Conclusions:**

Over the study period, health facilities made several modifications ART interventions to improve fit with their resource-constrained settings thereby promoting long-term sustainability. Further research evaluating the effect of these modifications on patient outcomes and ART delivery costs is recommended. Our findings have implications for the sustainability of ART scale-up programs in Uganda and other resource-limited settings.

## Background

Universal access to antiretroviral treatment (ART) is taking on increasing importance as a global public health priority. In November 2015, WHO released new ART treatment guidelines recommending that all diagnosed as HIV positive be enrolled on sustained ART regardless of disease stage [1]. In September 2015, the Sustainable Development Goals (SDGs) retained the goal of universal access to HIV treatment in the new international development agenda [2].

In sub-Saharan Africa (SSA), the region with the highest HIV/AIDS burden in the world, the acceleration in access to ART has depended substantially on external donor support principally through The President’s Emergency Fund for AIDS Relief (PEPFAR) and The Global Fund [3]. PEPFAR and The Global Fund supported a rapid expansion in ART coverage in SSA in an approach later known as WHO’s public health approach of ART roll-out to the largest number of HIV-positive people possible [4]. There are indications that international investments for ART scale-up are undergoing a slowdown and even declining according to some estimates [5–7].

Against this backdrop, the sustainability of ART scale-up programs has come into critical focus [8, 9]. Devising service delivery models that are suited to resource-limited contexts is acknowledged as an important strategy for fostering the sustainability of ART scale-up programs in SSA [10, 11]. Service delivery design alternatives to the traditional model of physician-centered, clinic-based care are becoming critical in SSA [4, 12, 13]. Sustaining and expanding ART coverage in resource-limited settings requires modifications and adaptations of ART delivery models to meet the continually rising demand [10, 11].

To promote sustainability, it is critical to systematically document the types of modifications made to ART service delivery models, the reasons for the modifications, and the extent to which they are common within health facilities to inform implementation strategies [14]. Because of the nature of funding for research, many studies tend to focus on the initial uptake of interventions with less empiric attention devoted to later-stage program development in implementing organizations [15, 16]. This study aligns with the literature suggesting that when interventions are implemented in organizations they undergo “life cycles” or phases of development which includes adoption, implementation, and sustainability [17, 18].

Modifying or adapting interventions from their original form to suit the context of provider organizations during the process of implementation has been linked with increased likelihood of sustainability in previous studies [17, 19–22]. These studies considered adaptation as one, among a set of strategies, adopted by implementing organizations to foster program sustainability. A seminal review by Stirman et al. [23] observes that “most studies that we reviewed did not describe adaptations or examine their impact on health-related outcomes.” They call for research that documents the types of modifications made to interventions and the contexts underpinning such modifications. Proctor et al. [15] highlight the relationship between adaptation and sustainability as one of the priority areas where additional research is recommended to advance knowledge on sustainability. They further note a paucity of evidence on the sustainability of interventions in low-income settings.

Within the field of antiretroviral therapy (ART), previous studies have examined modifications to ART focusing on specific components, such as personnel. To address the shortage of physician cadre during initial ART roll-out, many previous studies examined the role of task shifting from physician to non-physician cadre such as nurses [24–26]. There are few studies which have systematically documented spontaneous modifications to ART to understand program sustainability at the organization level of health facilities. The objective of this study was to identify the nature of modifications made to ART service delivery models by health facilities in Uganda to facilitate long-term sustainability (2004–2014).

Stirman et al. [27] proposed a classification system for reporting the various types of modifications made to interventions. Their scheme for coding modifications to interventions was derived from a systematic review and previous theoretical frameworks [27]. Within this classification system, “contextual modifications” are distinguished. They are defined as “changes made to delivery of the same program content, but with modifications to the format or channel, the setting or location in which the overall intervention is delivered, or the personnel who deliver the intervention” [27].

### Health systems background of Uganda

Uganda is listed among countries with a high HIV/AIDS burden. In 2015, an estimated 1.6 million people were living with HIV [8]. The demand for ART in Uganda is on the rise. In 2012, the national HIV prevalence rate was shown to have increased from 6.3% in 2005 to 7.4% in 2011 [28]. Uganda together with Nigeria and South Africa accounted for more than half of new HIV infections in SSA in 2013 [29].

Uganda embarked on an emergency ART scale-up program in June 2004 with high levels of dependence on external donor support. ART interventions were introduced on a trial basis in form of demonstration projects at select regional referral hospitals, with a phased expansion to primary health care facilities [30]. In the case of PEPFAR, a principal donor in Uganda, priorities are set for implementing health facilities in annual Country Operational Plans (COP). Uganda has an under-resourced health system [31]. It is listed by the WHO among countries with a severe health workforce shortage, with a low physician to population ratio [32]. Health worker morale is low as manifested in widespread absenteeism [33]. Supply chain bottlenecks for medicines and commodities are common [34]. Long distances to the nearest health facility are typical [35]. Dependence on donor aid in the health sector has been a persistent challenge [36, 37]. These characteristics of Uganda’s health system are shared with the majority of countries in sub-Saharan Africa [38, 39]; hence, innovations in ART service delivery models in Uganda have immense potential for adoption and implementation in the rest of SSA.

Studies are needed to understand how health facilities in Uganda have modified and adapted ART programs to achieve sustained delivery of ART services. This paper is derived from a larger study examining the sustainability of ART programs in health facilities in Uganda [30, 33, 37].

## Methods

### Research design

A mixed methods sequential explanatory research design was adopted. This entailed two study phases applied sequentially [40].

In the first, quantitative phase, a survey of ART clinic managers in a national sample of health facilities in Uganda was conducted. The goal of the quantitative phase was to assess the extent to which modifications to the ART program were prevalent and the most common types of modifications reported. In the second, qualitative phase, six health facilities were purposively selected for in-depth study. The goal of the qualitative phase was description and explanation of the nature of modifications made to ART programs by health facilities. We aimed to explore the motivations for and the contexts underpinning the modifications to ART service delivery models.

### Study sites and selection

The study sample comprised of 195 health facilities in Uganda which were accredited to provide ART between 2004 and 2009. This period is regarded as the emergency national ART roll-out phase [34]. Participating health facilities were drawn from the different levels of care of the Ugandan health system which ranged from the tertiary level (National and sub-national referral hospitals), secondary level (district hospitals), and primary level (county and sub-county health centers and clinics) [30, 41].

Health facilities were selected in a two-stage process. In the first instance, we obtained the published Uganda Ministry of Health, ART Unit Monitoring report, of March 2010. The report contained a list of 394 health facilities which were accredited to provide ART between 2004 and 2009. The list of accredited ART sites served as the sampling frame for the study. Health facilities were categorized as public, private-for-profit, private-not-for-profit, and HIV research clinics.

In the second stage, a sample of 195 (out of 394) health facilities was selected. The 394 health facilities were placed in 10 strata based on their location within Uganda’s 10 geographic sub-regions as designated by The Uganda Bureau of Statistics [42]. Through random sampling, health facilities were selected from each of the 10 strata based on proportionate representation. The detailed sample size calculation and final selection criteria of the 195 health facilities are described elsewhere [33].

In the second phase of the study, six health facilities were purposively selected for in-depth examination. Table 1 shows that at least two health facilities were selected from each of the three major health facility ownership categories (public/private-for-profit/private not-for-profit). The second selection criteria also included setting (rural/urban) and levels of care in the Uganda health system; large hospitals (PUB-001, PNF-002, PUB-002), midsize health facilities (PFP-002), and smaller health facilities (PF-001, PF-002). The final selection criteria sought to ensure that the selected health facilities were broadly representative of the modifications reported by providers in the first study phase. Specifically, the selected health facilities were those which (i) reported that ART had undergone major modifications (ii) endorsed the three most common forms of modifications (task shifting, spacing ART clinic appointments, modifying ART enrollment criteria) across the health facility survey.

**Table 1:** Characteristics of health facilities selected for in-depth study

Ownership	Acronym	Setting	No. of ART patients (June 2010)	No. of ART patients (June 2015)	% increase in patient load
Public	PUB-001	Urban	9540	24,408	61
	PUB-002	Urban	6358	7852	19
Private-for-profit	PFP-001	Peri-urban	84	19	−342
	PFP-002	Rural	166	478	65
Private not-for-profit	PNF-001	Urban	2556	4337	41
	PNF-002	Rural	324	612	47

### Data collection

#### Quantitative

##### Study instrument

A pre-tested questionnaire comprising 35 items was used to examine factors identified in the literature as potentially influential on program sustainability [16]. The questionnaire generated quantitative data relating to the characteristics of the health facilities such as the ownership category, the number of ART intervention components offered, setting (rural/urban) as well as respondent demographics. The questionnaire was aimed at generating data relating to the extent to which modifications to ART were prevalent in the 195 participating health facilities and the most common forms or categories of modifications reported between 2004 and 2014.

The questionnaire contained six items inquiring into the modifications made to ART by health facilities in Uganda (Table 2).

**Table 2:** Survey responses

Item	Setting	Responses *n* (%)				
1. Modifications to the ART program since commencement	Rural	28	(15.14)			
	Peri-urban	86	(46.48)			
	Urban	71	(38.38)			
	Total	185	(100)			
2. Health facilities reporting at least three modifications to ART: (a) Task shifting to non-physicians (b) ART clinic reduced the frequency of visits of some patients (c) ART clinic modified ART enrollment criteria	Rural	12	7.0			
	Peri-urban	82	49.3			
	Urban	72	43.7			
	Total	166	100			
3. Task sharing with non-physicians	Rural	27	14.92			
	Peri-urban	92	50.83			
	Urban	62	34.25			
	Total	181	100			
4. Expert patients assigned duties within the ART clinic	Rural	5	4.35			
	Peri-urban	21	18.26			
	Urban	89	77.39			
	Total	115	100			
5. Evolution of ART service delivery at health facility (*n* = 195)		No change	Minor modifications	Major modifications		
	Rural	00	01	28		
	Peri-urban	01	15	72		
	Urban	06	15	57		
	Total	07 (3.59%)	31 (15.90%)	157 (80.51%)		
6. Cadre of health workers represented in ART clinic leadership (*n* = 195)		Doctor	Clinical officer	Nurse	Midwife	Other
	Rural	06 (13.63%)	12 (18.8%)	26 (36.12%)	5	11
	Peri-urban	11 (25.00%)	38 (57.58%)	23 (31.94%)	1	5
	Urban	27 (61.36%)	16 (24.24%)	23 (31.94%)	2	3
	Total	44 (100%)	66 (100%)	72 (100%)	8	19

During on-site visits, a hard copy questionnaire was handed to the nominated respondent at each of the 195 health facilities. Typically, the nominated respondents were ART clinic managers who had been in service during initial implementation of ART services at their health facilities.

The completed questionnaire was collected from the respondents the following week on an ART clinic day to enable verification of ART program characteristics. Data were collected between January and April 2014.

#### Qualitative

In the second study phase, face-to-face interviews (*N* = 18) were conducted with three respondents from each of the six selected health facilities between February and June 2015. The respondents were the head of the ART clinic and two clinicians with the longest experience at the ART Clinic. Annual reports and websites of the selected health facilities were also reviewed to augment interviewee data.

### The interview guide

A semi-structured interview guide was constructed based on the results from the survey phase. The interview guide was designed with the aim of exploring the types of and broader contexts underpinning modifications to ART delivery models between 2004 and 2014. The interview guide consisted of 12 questions. A sample of the questions posed include (a) Over the past ten years, has your health facility made any modifications to the way ART is delivered? (b) What was the nature of the modifications made to ART programs, if any? (c) Why did your health facility make modifications to ART programs?

### Data analysis

#### Quantitative

Data generated from the quantitative questionnaire was edited, cleaned, and initially entered into Epi Data software (Version 3.1). Data were later exported into STATA software (Version 11.0). Descriptive statistics were generated with respect to provider organization characteristics, the extent to which modifications to ART were reported within health facilities and the most common types of modifications reported.

#### Qualitative

The interviews with ART clinic managers were audio-taped and later processed into text transcripts. As a first step, two authors (HZ and FS) separately read the transcripts multiple times for data familiarization [40]. The two authors then inductively coded the interviewee data manually.

In the second stage, the resulting codes were merged following a consensus process involving two authors. We then used a deductive approach by assigning codes to an existing classification system for reporting modifications to interventions proposed by Stirman et al. [27]; we adopted the four broad classifications relating to contextual modifications to interventions, namely (i) format (ii) setting (iii) personnel, and (iv) population. In this study, Format was defined as changes in the original mode of delivery of the intervention. Setting was defined as changes in the location where the treatment was offered. Personnel was defined as changes in the cadre of health workers offering the treatment. Population was defined as changes in the sub-population targeted from what was initially planned [27]. In the third stage, the resulting code categories from the second stage were grouped under four themes (format, setting, personnel, and population) [43] following a team-based process involving all the authors.

### Integration of qualitative and quantitative data

Core qualitative analyses were undertaken based on the four broad classifications of contextual modifications to interventions [27]. Quantitative data were used for expansion of the four study themes [40, 44].

## Results

### Characteristics of study population

There were 195 health facilities which participated in the study. In terms of ownership, 121 (62%) were public facilities compared to 35 (18%) were private not-for-profit, 33 (16%) which were private-for-profit, and 6 (3%) which were HIV Research clinics.

Almost half of the health facilities 88 (45%) were located in peri-urban settings (urbanized rural areas) compared to 78 (40%) which were in located in urban settings and 29 (15%) which were based in rural settings.

Table 3 shows that nearly half of all participating health facilities (47%) started offering ART between 2005 and 2006.

**Table 3:** Year when ART scale-up was implemented at participating health facilities

Year ART started	Number of health facilities	Percentage
2004	41	21
2005–2006	92	47
2007–2008	41	21
2009	21	11
	195	100

The average age of the respondents was 36 years, ranging from 22 to 59 years. The average number of years spent at the health facility was 5 years.

In Table 1, the characteristics of the six health facilities selected for in-depth examination of contextual modifications made to ART over a 10-year study period (2004–2014) are presented.

### Contextual modifications to art interventions by provider organizations

The results of the survey and interviews are presented in four sections representing the four broad themes proposed for classifying contextual modifications to interventions [27]. The quantitative and qualitative findings are integrated and presented together under four themes [40].

### Format

The results from the survey of health facility representatives show that 185 (95%) of health facilities made modifications to the format of ART delivery between 2004 and 2014.

When health facility representatives were asked the question: Which of the following most accurately describes how ART service delivery has evolved at your health facility since it was originally implemented?

The majority of health facilities 157 (81%) selected the “undergone major modifications” option.

Three forms of modifications to ART were reported by 85% of the health facility representatives between 2004 and 2014. These include (i) task shifting to non-physicians (ii) reducing frequency of clinic visits, and (iii) modifications to ART enrollment criteria.

Across the 18 interviews with ART clinic managers from the six health facilities selected for in-depth study, substantial modifications to traditional ART delivery models were reported as a strategy for long-term intervention sustainability.

#### Reducing the frequency of clinic appointments

The results demonstrate that ART-providing organizations reduced the frequency of patient clinic appointments from once every 3 months to once every 6 months for patients who were deemed clinically stable. Interviewees justified modifications to Uganda’s national ART guidelines, with regard to patient review periods, as motivated by the need to decongest ART clinics.

The moment someone has started on ART and they have stabilized, they don’t have to come here and sit down the whole day because guidelines dictate that every three months they have to come in. We give appointments that are appropriate for us to avoid congesting the place [Interviewee 1, PNF-001].

ART clinic days were designated days of the week for review of patients enrolled on ART. Interviewees described the out-patient loads on ART clinic days as overwhelming. Table 1 shows that in three of the six health facilities, patient volumes nearly or actually doubled. Health facilities were overwhelmed by the large patient volumes as the workforce was over stretched and physical space became inadequate.

We reached a point when the clinic was terribly congested. My staff were overwhelmed by the patient numbers. In fact, we did not even have enough physical space to accommodate the patient queues and all who sought care here [Interviewee 2, PFP-002].

Table 1 shows increases in patient volumes in all but one of the health facilities selected for in-depth study.

Coping mechanisms for managing outpatient burdens were devised through introducing a system of rationalizing visits to the ART clinic.

#### Pharmacy-only refill programs

At three of the health facilities we selected for in-depth study, a pharmacy-only refill program was introduced for patients who were deemed clinically stable. Patients were deemed clinically stable by clinicians if they had a high CD4 count and a viral load that was not detected. These patients were exempted from routine reviews as required under Uganda’s national ART treatment guidelines of 2008 by the attending clinician. Stable patients were only required to pick drugs (ARVs) “refills” at the medicines dispensing units within the ART clinic.

We modified the guidelines somewhat. For patients who have been on ART and are stable, with a high CD4 count and a viral load that is not detected, all they do is come in for a refill of ART medicines [Interviewee 2, PUB-002].

These measures had the effect of reducing patient queues to see clinicians. They were also reported to have helped in decongesting ART clinics and aided in the optimization of clinician time for more clinically demanding HIV cases and other non-HIV services.

Interviewees revealed that the pharmacy-only refill program was implemented as a mitigation measure in response to the increase in patient volumes. Respondents from two of the six health facilities indicated that at least a third of patients in the ART clinic were on the pharmacy-only refill program. The lack of physical space to accommodate the rapidly expanded patient volumes was highlighted as another motivation for clinic decongestion by health facilities.

The finding that the pharmacy-only refill program was initiated principally to decongest the ART clinic was also confirmed through document analysis with respect to the health facilities. An annual report in one of the health facilities reported the pharmacy-only refill program as their own innovation in ART service delivery.

### Setting

According to the classification of contextual modifications we adopted [27], setting was defined as a change in the location of where a treatment was originally offered at initial implementation. Our findings demonstrate that providers sought to promote non-clinic-based delivery platforms. Interviews with ART clinic managers revealed that there were costs associated with each client visit to the ART clinic. As such, deliberate strategies were reported to scale down on facility-based care to reduce the overall costs of ART delivery at the health facilities. The strategies devised are described below.

#### Home-based care program

In a mission hospital and a private not-for-profit health facility selected for in-depth study, a home-based care program was introduced for patients who lived within the neighborhood of the hospital. A mobile team comprising lower cadre staff from the hospital visited patients in their homes to make reviews and dispense drugs for patients who were deemed clinically stable. The mobile team was led by a nurse and comprised “expert patients” and counselors who had attended a 2-week training prior to joining the mobile teams.

We introduced an outreach program where we visit stable clients in their homes in the areas neighboring the hospital so those don’t have to come here. We visit them in their homes to see how they are faring [Interviewee 3PNF-002].

The home-based program was implemented for patients whose homes were within a radius of 15 km from the health center. Modifying the setting of the ART delivery from a facility-based to a home-based one was motivated by the mutually re-enforcing goals of decongesting the ART clinic and enhancing the convenience of patients deemed clinically stable. The home-based care teams were led by lower cadre and “expert clients” who were co-opted in these teams. Those selected as expert patients were those who had been enrolled on the ART program for a period of at least 5 years, had a minimum of secondary school education, and exhibited a passion for helping out in the clinic and counseling peers.

#### Patient information system-based reviews

In contrast to ART standard of care which is facility-based and involves visits to the ART clinic by patients for scheduled review appointments, a modification was devised at a public hospital and not-for-profit health facility involving patient reviews based on data in the electronic databases. This was only applied for a selection of patients deemed clinically stable. On the basis of patient outcomes, a selection of patients was reviewed by phone call. Patient records in the health facility’s electronic databases were then updated accordingly.

We look up the patient file, call the patient and find out how they are doing while we are filling the file. You know they just don’t want to come and sit here [Interviewee 3, PNF-001].

This substitution in the standard of care model was partly driven by the need to adopt a more patient-centered approach which at the same time promoted the service delivery goals of the provider.

A private not-for-profit hospital representative recounted a situation where a donor grant, which was their principal source of funding for ART service delivery, was withdrawn. This prompted cuts in staffing levels and an increased reliance on HIV treatment information systems in ART services delivery to account for cuts in staffing. The results demonstrated that modifications to ART interventions were partly prompted by the need by health facilities to maintain ART delivery even in the event of loss of funding from external donors.

### Personnel

#### Task sharing with non-physician cadre

Of the health facility representatives surveyed, 181 (93%) reported task shifting of ART management tasks from physician to non-physician cadre. The range of tasks shared with non-physician cadre included clinical management of ART which, under standard of care guidelines, is physician-led. The cadre of health workers enrolled in ART management roles included clinical officers, nurses, midwives, and expert clients. Clinical officers attain a post-secondary school training of 3 years at a tertiary non-university institution.

The results show that task sharing was not only at the clinical level but also extended to leadership roles such as ART program team leaders at participating health facilities. Table 2 shows that clinical officers were the most represented cadre in ART clinic leadership in our sample of health facilities. This was followed by nurses and medical doctors. Table 2 further illustrates that the majority of doctors in ART clinic leadership were based in health facilities in urban areas.

#### Nurse-centered care models

The representatives of a public hospital and a mission hospital indicated that their health facilities adopted deliberate nurse-centered ART delivery models to align with the shortage of physicians. The range of task sharing by nurses was broad.

There is a lot of task shifting here. Every nurse here is a dispenser, a counselor, a triage nurse, she can work in the laboratory and can do phlebotomy, do an HIV test, work in a treatment room and work in a Tuberculosis room [Interviewee 2, PUB-001].

Capacity-building programs for nurses were implemented to equip them for an expansion of their traditional scope of practice. Table 4 highlights nurse capacity-building strategies a private not-for-profit hospital.

**Table 4:** Nurse capacity-building program at PNF-001

Program	Regularity
Weekly continuing medical education sessions for nurses	Wednesdays
Nurses staff development seminars	Bi-weekly
In-house simplified ART treatment protocols for nurses	Manual updated periodically
Off-site ART refresher trainings	Variable

#### Use of “expert clients” in staffing roles

Of the 195 health facilities, 115 (59%) reported that long-term patients known as “expert clients” were co-opted into staffing roles within their ART clinics. Table 2 shows that the majority of health facilities reporting a role for “expert patients” in ART clinics were based in urban areas. ART clinic managers described ART delivery as labor intensive requiring staffing levels commensurate with the large patient volumes. They justified the role of expert clients as necessary in the context of ART workforce shortages.

We have expert clients who do a lot of work here including managing patient queues, peer adherence counseling and pill counting. We give them some little incentives to boost their morale [Interviewee 3, PUB-001].

Expert clients helped plug staffing shortages especially in non-clinical tasks in the health facilities we examined.

### Population

Stirman [27] defines the theme of population as relating to “An intervention that was specifically developed to target a particular population is being delivered to a different population than originally intended.” Modifications to the public health approach to ART roll-out that was emphasized between 2004 and 2005 were observed [4]. Health facilities adopted the strategy of scoping service zones and capping patient numbers which are described in more detail in the following sections.

#### Scoping of service zones

Interviews with ART clinic managers revealed that during the initial ART roll-out phase, increased enrollments on ART were the priority of external donors. Interviewees indicated that donor priorities shifted over the course of ART implementation. In addition to patient retention, ART donors set patient cohort adherence targets for provider organizations. One of the performance benchmarks set was reducing the number of patients lost to follow-up.

To align with patient adherence targets set by donors, two urban for-profit clinics re-defined the population they targeted based on the distance between the patients’ home and the health facility. This was also in keeping with a more patient-centered approach of enhancing client convenience such as targeting patients for whom transport costs would be less of a barrier to ART adherence.

After two years of implementing ART services, our patient numbers grew tremendously. We began getting clients from districts miles away from here. However, patient follow-up for those in far-flung districts was a challenge for us and the patients because of transport costs. As a result, I told my staff to focus on patients who lived within a 5 kilometer radius [Interviewee 2, PFP-002].

#### Capping of patient numbers

In some of the health facilities, there were deliberate attempts to cap the number of patients enrolled on the ART program to align with the service delivery capacity of providers. One of the health facilities reported that patient volumes outstripped their staffing strength necessitating a limit to the number of patients to be enrolled on the program.

In 2011, we decided to set a limit to the number of patients we could enroll to about 40 a year. This was because we were overwhelmed by the number of patients who came here for treatment [Interviewee 2,PFP-001].

Table 1 shows the growth in patient numbers at each of the six health facilities. The capping of patient numbers was attributed to the dramatic increase in patient numbers at the health facilities.

### Summary of motivations for modifications to ART service delivery models

Table 5 lists the four themes of contextual modifications, the modifications identified under these themes, the reasons elicited for the modifications, and the level at which the modifications were affected.

**Table 5:** Summary matrix of modifications made and reasons

Classification	Modification type	Reason for modification	Level modification initiated
Format	Reducing the frequency of clinic appointments	Decongesting ART Clinics	Provider organization level/clinician
		Optimizing clinician time and expertise	
	Pharmacy-only refill program	Decongesting ART clinics	Provider organization
		Cost reduction	
Setting	Home-based care program	Patient convenience	Provider organization
		Decongesting ART clinics	
	Information system-based reviews	Loss of principal funding from donor	Provider organization level/clinician
		Patient convenience	
Personnel	Task sharing with non-physician cadre	Shortage of physician cadre	Provider organization
	Nurse-led care model	Inability to attract physician-level cadre	Provider organization
	Use of “expert clients”	Labor intensive nature of intervention	Provider organization
		Staffing shortages due high ART demand	
Population	ART clinic sub-population designation	Rationalizing visits to clinic	Provider organization/clinician
	Scoping of service zones	Align with new donor priorities	Provider organization/clinician
		Patient convenience	
	Capping patient numbers	Align with delivery capacities:	Provider organization
		(a) Staffing (b) physical space	

The table illustrates that decongesting ART clinics was repeatedly cited by health facilities as a motivation for modifying the existing ART service delivery models. The goal of decongesting ART clinics cuts across two classifications of modifications (setting and format). Table 5 illustrates that ART clinic decongestion was prompted by insufficient physical space to accommodate the rapid increase in patient volumes. Table 2 illustrates that the nearly half of all participating ART clinics were based in peri-urban areas.

Human resources for health (HRH) constraints were prominent in the factors elicited by health facilities for the extensive modifications to ART service delivery models. Table 5 shows that HRH constraints were the most cited group of factors reported by health facilities as motivation for modifying ART service delivery models and cut across almost all the four themes of contextual modifications. The specific HRH constraints cited included the need to reduce the workloads of the overburdened workforce and the need to optimize the available clinician expertise given the shortage of physician cadre at the sampled health facilities. The labor intensive nature of ART service delivery prompted innovations in expanding labor supply on part of the providers by co-opting non-physician cadre such as nurses and midwives in clinical roles and “expert clients” in filling staffing shortages.

The need to adopt a more patient-centered approach in ART service delivery influenced modifications in standard of care (soc) approaches. Table 5 shows the motivation for switching setting to home-based care, and the increased reliance on patient databases to conduct reviews was intended to ease burdens on patients deemed as “stable.”

Table 5 reveals that donor policies and shifts in priorities were an external influence on the decision by health facilities to modify service delivery mechanisms. Interview data shows that during initial ART scale-up, mass enrollment was the key benchmark set by donors. However, during the course of implementation, donors switched provider performance criteria to include reducing lost to follow-up cases. This new orientation prompted a switch in focus from the WHO public health approach to ART scale-up in favor of a scoping of service zones to align more closely with donor criteria and hence ensure sustainment of external grants.

Table 5 illustrates that the majority of modifications were made at the organizational level of health facilities such as by the ART program leadership as compared to individual clinicians.

## Discussion

We used a mixed methods approach to identify modifications to ART service delivery models by health facilities in Uganda to foster program sustainability between 2004 and 2014. Our findings show that the majority (95%) of health facilities reported making modifications to ART delivery models during the study period. ART interventions were modified from the standards stipulated in Uganda’s national ART treatment guidelines. The modifications were made with respect to all the four classifications of contextual modifications suggested by Stirman et al. [27], namely, format, setting, personnel, and population. There were deliberate strategies to scale down clinic-based care by providers through reducing the frequency of clinic appointments. To realize this strategy, the ART clinic population was divided into two based on those deemed clinically stable and those not. A range of innovations in service delivery were reported by health facilities. These included the introduction of “pharmacy-only refill” and home-based care programs in attempts to decongest ART clinics and reduce the overall costs of ART delivery. Task shifting of ART delivery tasks from physicians to relatively available non-physician cadre was reported in 181 (93%) of participating health facilities.

The majority of the modifications made to ART by provider organizations were with respect to service delivery mechanisms and were related to how, where, and to whom the intervention would be delivered. The literature on health program sustainability has referred to this distinction as one between the core components and the customizable components of interventions [15–17, 23]. Hirschhorn et al. [13] have called for an appropriate balance between adaptation and flexibility in the scale-up of HIV interventions [46]. In contrast with a previous study [45], we found that most modifications to ART were made at the organization level as opposed to individual clinicians.

### Motivations for modifications to ART service delivery models

Interviews with ART clinic managers suggest that the modifications to ART service delivery models were prompted by the resource-constrained operational contexts at participating health facilities. Our findings further demonstrate that the demand side (patient case loads) outstripped the “supply side” or the service delivery capacities of participating health facilities which prompted modifications in the existing ART service delivery models. A range of mitigation measures were devised by health facilities. These included decongesting ART clinics through reducing the frequency of clinic appointments. Uganda’s national ART treatment guidelines required review of patients every 3 months [47]. Providers extended this period from 3 to 6 months for patients determined to be clinically stable. Additionally, non-clinic-based care platforms were devised by health facilities. Previous studies have documented innovations in decongesting ART clinics under quasi-experimental settings [11, 48–50]; our study reports spontaneous modifications to ART delivery models by health facilities in Uganda.

The summary of the reasons for modifications to ART service delivery models illustrated in Table 4 demonstrates that human resources for health constraints are a major motivation for modifications in service delivery models. The shortage of physician-level cadre at participating health facilities prompted task shifting of ART clinical tasks to midcadre such as nurses and midwives and a reliance on “expert” patients to plug staffing shortages. The role of “expert” patients in HIV services delivery in resource-limited settings has been reported in a number of studies [51, 52]. The majority of health facilities reporting a role for “expert patients” were based in urban areas. A population-based study in Uganda found that patients often bypassed nearer health facilities in rural areas and sought care in large hospitals which are predominantly based in urban areas [35]. It is plausible that expert patients are more pronounced in urban facilities such as at district and regional referral hospitals which have relatively high patient volumes and workloads [37]. Furthermore, health facilities reported a need to optimize the available clinician workforce through non-clinic-based care platforms. There have been numerous studies reporting on task-shifting approaches to facilitate ART scale-up [14, 24–26, 32]. However, beyond routine service delivery, our study suggests that non-physician cadre was co-opted into leadership roles within ART clinics as reflected in Table 2. Alleviating HRH constraints through adaptations in service delivery models is widely acknowledged as in important strategy for the sustainability of ART scale-up in resource-limited settings [10, 11, 33, 50].

Our findings demonstrate that health facilities sought to modify their service delivery models to align with a more patient-centered approach. Health facilities actively sought to enhance the convenience of patients through reducing the time spent at clinics through spacing appointments for routine reviews and fast-track drug refill programs. This informed alternatives to clinic-based care such as home-based care programs and review by phone calls for patients deemed stable. The costs associated with more frequent visits to ART clinics in terms of transport and time have been highlighted by previous studies [10, 11, 35].In this connection, there have been mounting calls for reframing HIV treatment towards more patient-centered approaches [10, 11].

Some of the modifications were externally influenced such as through the withdrawal of grants by external donors and shifts in donor priorities and policy during the course of ART implementation. A review article by Scheirer [17] found that modifications were made to interventions ranging from mental health to heart health to align with changes in the priorities of donors in the USA and Canada. The finding that the broader environment underpinning the implementation of interventions influences program sustainability is supported in the literature [53–55]. The broader environment is understood to refer to factors that are external to an implementing organization. This includes variables such as donor priorities, policy frameworks, and the favorability of the socio-economic and political environment for the sustainment of an intervention [16, 53, 54].

Our findings highlight the utility of a “systems thinking” lens in understanding health service delivery dynamics in resource-limited settings [30, 34, 53]. This study suggests a dynamic interaction in the building blocks of the health system in the way they influence ART service delivery at the local, facility level [38, 56]. This was especially in regard to the way resource input shortages (human resources for health, financing) affected ART service delivery and necessitated modifications to traditional delivery approaches by health facilities in response to an under-resourced health system.

### Implications for ART program sustainability

With the growing importance of universal access to HIV treatment in the global health and development agendas, our findings add to the evidence on alternative ART service delivery models for realizing ART scale-up goals. In this regard, our findings add to previous efforts by Bemelmans et al. [11], Decro et al. [50], and Lazarus et al. [14]. The contribution of the present study is in showing how prevalent and what the most common types of modifications to traditional ART delivery models are, in a relatively large sample of health facilities in Uganda.

Babigumira et al. [57] found that pharmacy-only refill programs were more cost effective than standard of care (SOC) approaches in a large urban clinic in Uganda [14]. Findings by Jaffar et al. [48] suggest that home-based care programs are worthy alternatives to clinic-based platforms in resource-poor contexts [50]. Additionally, from the perspective of patients, non-clinic-based platforms have been associated with savings in costs and time [10, 14].Our study’s contribution to the foregoing studies is in documenting *spontaneous* modifications to ART delivery mechanisms by health facilities under non-experimental conditions. This study adds to the literature on task sharing with non-physician cadre in resource-limited settings by placing it within a broader set of modifications to ART aimed at fostering the sustainability of ART programs [24–26].

The study has implications for broader health service delivery goals in low-income countries. In efforts to respond to the rising non-communicable diseases (NCD) epidemic, lessons on task shifting to trained non-physician cadres, and the use of select long-term “expert” patients in countries with critical shortages in health human resources could potentially alleviate overflowing outpatient clinics for treating diabetes, cardiovascular diseases, and cancers in resource-limited settings. Our findings add to efforts by Rabkin et al. [12] and Letebo et al. [58] in calling for the leveraging of ART scale-up lessons in response to the NCD pandemic. Our study elicits provider innovations in chronic disease care management that have potential for implementation in resource-limited settings.

### Implications for ART program managers, policy makers, and future research

In light of the motivation by health facilities to reduce the costs of ART delivery through modifications to standard of care (SOC) protocols, further research evaluating the impact of these adaptations on patient outcomes and ART delivery costs is recommended. A study conducted in Malawi, South Africa, Mozambique, and Congo (DRC) reported that community-supported ART delivery models showed good patient outcomes for patients stable on ART [11]. However, this study concludes with a call for more rigorous research on the effect of these innovations on patient outcomes.

We further note that some health facilities reported a focus on patients who resided in areas with closer geographical proximity to them. This was reportedly aimed at aligning with donor targets on patient follow-up. While contraction of coverage is understandable from the perspective of providers, a more balanced approach is warranted. It is important to ensure that adaptations in ART service delivery are not at odds with the commitment “to leave no one behind” as enshrined in the Sustainable Development Goals (SDGs) and the coverage ideals emphasized in universal health coverage (UHC).

Providers reported extensive modifications in ART delivery models especially with regard to stable patients. Further research assessing the quality of care of patients in health facilities which report adaptations and modifications in ART delivery models is recommended.

The study findings provide input to national and international bodies that set ART treatment guidelines especially with respect to resource-limited settings. Our findings show that health facilities evolved criteria for reducing the frequency of visits to the ART clinic based on clinical assessment of individual patients. This was reported to have helped in decongesting ART Clinics, optimized clinician time, and perceived to have reduced the overall costs of ART delivery by providers. Scaling-up these provider modifications could go a long way in reducing outpatient burdens in ART clinics in resource-limited settings. Specifically, devising a framework for reducing the frequency of visits to the ART clinic based on the clinical needs of individual patients is worthy of consideration in revising protocols and related policies for resource-limited settings. Our study adds to a recent call by Duncombe et al. [10] for differentiated care models.

Increased international investments in further ART scale-up in sub-Saharan Africa are, undoubtedly, a top priority in global public health. Indeed, many previous studies have focused on this dimension of financial sustainability [3, 5–7]. Our findings suggest, however, that modifications and adaptations to traditional ART delivery mechanisms could potentially ease the mounting fiscal and resource pressures for meeting the enormous buildup in demand for ART. This is especially topical following World Health Organization’s 2015 treatment guidelines which recommend that all diagnosed as HIV positive be enrolled on sustained ART regardless of disease stage.

### Limitations

We wish to acknowledge the following limitations to our study. Our principal data sources depended on self-report information such as the self-administered questionnaire and semi-structured interviews. We endeavored to mitigate this limitation by triangulation of data sources through on-site verification visits and document review. Given the retrospective nature of the study and that we examined the modifications to ART programs since 2004, recall bias was a possible limitation. We however relied on multiple informants per health facility to compare respondent data on timelines. In the second qualitative phase, we aimed for description of the modifications made to ART delivery mechanisms by health facilities. Hence, we did not aim for statistical generalization in our sample size and selection but sought an in-depth explanation of the findings from the survey in the first, quantitative study phase.

### Strengths

The adoption of a mixed methods approach was one of the strengths of this study. We sought a nationally representative sample of health facilities in Uganda which were accredited to provide ART between 2004 and 2009 to assess the extent to which modifications to ART were common. In the qualitative phase of the study, we explore the nature of modifications made to ART and the broader contexts underpinning the modifications. This study adds to the literature examining modifications and adaptations in ART service delivery models in resource-limited settings. We use a classification framework to systematically document these modifications and contribute to our understanding of their relationship with the long-term sustainability of ART interventions in health facilities in Uganda.

## Conclusions

Over the study period, health facilities made several modifications to ART interventions to improve fit with resource-constrained settings thereby promoting long-term sustainability. Further research evaluating the effect of the identified modifications on patient outcomes is recommended. The findings have implications for the sustainability of ART scale-up programs in Uganda and other resource-limited settings.
